# Generic, scalable and decentralized fault detection for robot swarms

**DOI:** 10.1371/journal.pone.0182058

**Published:** 2017-08-14

**Authors:** Danesh Tarapore, Anders Lyhne Christensen, Jon Timmis

**Affiliations:** 1 School of Electronics and Computer Science, University of Southampton, Southampton, United Kingdom; 2 York Robotics Laboratory and the Department of Electronic Engineering, University of York, Heslington, York, United Kingdom; 3 Bio-inspired Computation and Intelligent Machines Lab, Lisbon, Portugal; 4 Instituto Universitário de Lisboa (ISCTE-IUL), Lisbon, Portugal; 5 Instituto de Telecomunicações, Lisbon, Portugal; Chongqing University, CHINA

## Abstract

Robot swarms are large-scale multirobot systems with decentralized control which means that each robot acts based only on local perception and on local coordination with neighboring robots. The decentralized approach to control confers number of potential benefits. In particular, inherent scalability and robustness are often highlighted as key distinguishing features of robot swarms compared with systems that rely on traditional approaches to multirobot coordination. It has, however, been shown that swarm robotics systems are not always fault tolerant. To realize the robustness potential of robot swarms, it is thus essential to give systems the capacity to actively detect and accommodate faults. In this paper, we present a generic fault-detection system for robot swarms. We show how robots with limited and imperfect sensing capabilities are able to observe and classify the behavior of one another. In order to achieve this, the underlying classifier is an immune system-inspired algorithm that learns to distinguish between normal behavior and abnormal behavior online. Through a series of experiments, we systematically assess the performance of our approach in a detailed simulation environment. In particular, we analyze our system’s capacity to correctly detect robots with faults, false positive rates, performance in a foraging task in which each robot exhibits a composite behavior, and performance under perturbations of the task environment. Results show that our generic fault-detection system is robust, that it is able to detect faults in a timely manner, and that it achieves a low false positive rate. The developed fault-detection system has the potential to enable long-term autonomy for robust multirobot systems, thus increasing the usefulness of robots for a diverse repertoire of upcoming applications in the area of distributed intelligent automation.

## Introduction

Robot swarms have the potential to take on numerous real-world tasks [1]. In particular, tasks that require sensing or action over large areas or at a high spatiotemporal resolution, such as warehouse management, agriculture automation and environmental monitoring, are candidates for application of future swarm robotics systems. In many cases, however, a system must be dependable and display a high degree of tolerance to faults before it can be deployed in real-world scenarios.

The autonomous detection of faults in robotic systems is a challenging problem. For robot swarms operating in real-world scenarios, we can not rely on any external observatory infrastructure to directly detect presence of faults. Rather, the robots of the swarm have to employ a more localized solution and execute fault-detection software onboard. The software must rely on readings from the robots’ limited, imperfect sensors, to infer the presence of robots whose behavior deviates from the normal or expected behavior. Such fault detection systems can be divided into two categories: *endogenous fault detection* and *exogenous fault detection*. The aim of endogenous fault detection is to enable a robot to detect the presence of faults in itself. Several approaches to endogenous fault detection for autonomous robots have been proposed: in model-based approaches, the actual behavior is compared to the predictions of either a single model of normal behavior, or to a set of models of the robot in different operational states [2]. In data-driven approaches, normal behavior and faulty behavior are learned based on observations [3]. It has been demonstrated that endogenous fault detection approaches can enable a robot to detect the presence of faults such as broken sensors and actuators, see [3–6] for examples. However, catastrophic faults, such as a malfunctioning power source or issues with the onboard computational hardware usually cannot be detected endogenously as they render the robot completely non-operational.

In multirobot systems, robots are not limited to detecting faults endogenously: robots also have the opportunity to perform exogenous fault detection, that is, to detect the presence of faults in one another. Exogenous fault detection has the potential to detect any type of fault, including catastrophic faults. When endogenous fault detection and exogenous fault detection are combined, it is sometimes referred to as *multi-layered fault detection* [7].

For tightly coupled multirobot systems, in which coordination is based on negotiation and global knowledge, fault detection and fault accommodation can be an integral part of task allocation. In ALLIANCE [8], for instance, robots communicate or observe one another’s progress and allocate tasks based on priorities. Robots get *impatient* when there is a lack of progress in critical tasks, and robots that are unsuccessfully executing a task, potentially due to a fault, become increasingly likely to abandon the task over time. In market-based approaches [9, 10], unresponsive robots do not participate in task negotiation and are therefore not allocated to tasks, and robots underperforming due to faults may lose their assignment or be reassigned [11]. However, scalability and communication issues, along with the reliance on global knowledge, prevent such approaches from being applied in robot swarms.

In some of the simplest approaches to decentralized fault detection in robot swarms, robots periodically communicate, either explicitly [12] or implicitly [13], that they are still alive and operational. Absence of communication is indicative of a fault. Such an approach to fault detection in robot swarms is one of the few that have been demonstrated on real robotic hardware [13], but only complete failures (robot death) were considered.

Several model-based approaches to fault detection in decentralized multirobot systems have been studied. In [14], for instance, an approach in which distributed fault detection based on a bond-graph modeling framework for robots in a leader-follower formation was proposed. Residuals, defined as the difference between model-predicted and actual measurement outputs, were used for the detection. To compensate for the noise and model imperfections, actual trajectories were tracked using a scheme based on distributed, decentralized, extended Kalman filters [15]. Millard et al. [16] proposed a fault detection approach for robotic swarms, in which robots use onboard simulation to predict the behavior of nearby robots. Although the approach has not yet been demonstrated on a swarm of real robots, exogenous, forward simulation of behavior for a single real robot was studied in [17].

Different data-driven approaches to fault detection in multirobot systems have been proposed. In [7], for instance, robots that meet one another share their respective estimates on the relative distance and direction to one another. Based on a comparison, robots can infer the presence of faults in their onboard sonars and compass sensor. In [18], faults are inferred based on a comparison of task performance rather than on behavior or specific sensor readings. The rationale is that a fault would compromise a robot’s ability to perform the task, and consequently its individual performance. In the approach, robots record their individual performance and transmit it to nearby robots. To detect faults, each robot uses a statistical classifier to determine if its performance is an outlier with respect to the performance of recently encountered robots. In [18], the approach is assessed on robots performing a foraging task, and it is demonstrated that faulty robots are detected even in environments with heterogeneous resource distributions and in environments in which the concentration of forageable resources change over time.

Flexibility and adaptivity are important features in fault-detection systems, in particular, for systems deployed in unknown and changing environments. In tasks where frequent performance assessment is possible, local comparisons of performance indicators may be sufficient to detect faulty units due to their inferior performance, such as in [18] discussed above. However, when no easily quantifiable and frequently updated performance indicators are available, such approaches cannot be applied. Model-based approaches, such as [14], and Millard’s et al.’s simulation-based approach [16], are not applicable in scenarios in which the robots’ behavior changes over time due to adaptation or changing environmental conditions, given that detailed knowledge about correct robot behavior is assumed to be available prior to deployment.

A recently proposed approach to fault detection was presented in [19], where the presence of faults is inferred based on the detection of abnormal behavior. For the classification of normal vs. abnormal behaviors, the *crossregulation model* (CRM) [20–22] was used. The CRM describes the dynamics of effector T-cell populations and regulatory T-cell populations during interactions with antigen presenting cells. In the approach presented [19], behavior was represented as a fixed-length vector of binary behavioral features. The notion of normal and abnormal behavior was learned online, and it was demonstrated that the fault-detection system continued to display a high level of performance even when behaviors changed over time [19] and when displaying composite behaviors [23]. While promising, the approach has so far only been assessed in an simulated abstract toroidal environment, where the robots were idealized point-sized entities with unrestricted sensing capabilities.

In this paper, we present and study a fault detection approach based on CRM-based abnormality detection for robot swarms. The approach takes into account the limited sensing and actuation capabilities of a real swarm robotics hardware platform, the e-puck [24]. Our study presents three novel contributions, namely: (i) a distributed behavior observation model for large-scale spatially distributed robot swarms, that allow a robot to characterize the behavior of its neighboring robots using limited sensing capabilities and sporadic observations; (ii) fault detection that can be parameterized to balance the trade-off between latency in identifying faulty robots in the swarm, and the number of false-positive incidents; and (iii) a distributed swarm coalition algorithm to transcend the decisions made by individual robots on their neighbors to a robust swarm-level decision on the normal/faulty status of the robot, crucially required to provide collective fault-recovery strategies for the swarm.

## Materials and methods

**Fig 1:**
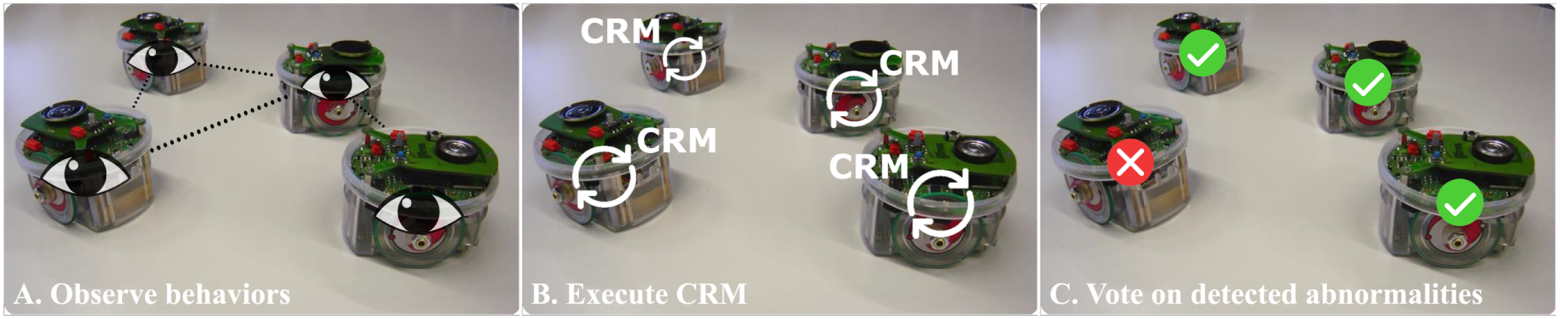
Overview of our distributed fault detection approach. A: Robots observe the behavior of their neighbors, and estimate the corresponding behavioral features. B: Each robot executes an individual instance of the CRM to detect abnormal behaviors in the observations. C: Robots of the swarm form voting coalition on the detected abnormalities to consolidate their individual-level decisions.

In this section, we describe our exogenous fault-detection approach for large-scale robot swarms (physical parameters of robot swarm in Table 1). The process of fault detection is divided into the following three phases (see Fig 1): (i) robots observe and characterize the behavior of their neighbors over a period of time, and estimate the corresponding behavioral features; (ii) every robot classifies the observed behaviors as normal/abnormal, where abnormalities are consequent to faults in the robot; and (iii) the robots form voting coalitions to consolidate their individual-level decisions on the detected behavioral abnormalities. The three phases of robot fault detection are described below.

**Table 1:** Parameters of the robot swarm.

Parameter	Description	Value
|*R*|	Number of robots in swarm	20
$|{\vec{v}}_{\text{max}}|$	Maximum linear speed of a robot	5 cm/s
$|\vec{v}|$	Linear speed of a robot	–
${\dot{\omega }}_{\text{max}}$	Maximum change in heading of a robot	1.9 radians/s
$\dot{\omega }$	Change in heading of a robot	–
*h*	Robot inter-wheel distance	5.3 cm
Δ*t*	Length of control-cycle	0.1 s
*W*_*s*_	Length of the time window for estimating change in heading of observed robot	5 s
*W*_*l*_	Length of the time window for feature-vector estimation of observed robot	10 s

### A. Robot behavior characterization

In the first phase of fault detection, every robot of the swarm executes Algorithm 1 (Phase A) at the start of each control cycle to observe the behavior of its neighbors over a period of time, and characterize their behaviors. Individual characterization is followed by an exchange of behavioral characterizations between neighboring robots, and a voting scheme to select the most likely characterization. The characterization of robot behavior is divided into three different classes: (i) the robot’s immediate environment (sensors), (ii) the robot’s actions (actuators), and (iii) the robot’s response to events (sensorimotor interactions). Behavioral features from each class are used to characterize an observed robot’s behavior, with each feature encoded in Boolean form (present = 1, absent = 0). Features are concatenated to form a binary string, the feature vector. In our simulations, a feature vector comprises the concatenation of six features (F1, F2, F3, F4, F5, F6), with two features from each class.

**Algorithm 1 A robot’s control loop (fault-detection algorithm).**

1: *time* ← 0 s

2: **while** *time* < *Experiment Duration* **do**

3:  Increment *time*

4:  {Phase A: Estimate behaviors of neighboring robots, using the range-and-bearing sensors}

5:  Estimate behavioral feature vectors of all observed robots (Eqs (1)–(19), Algorithm 2).

**Fig 2:**
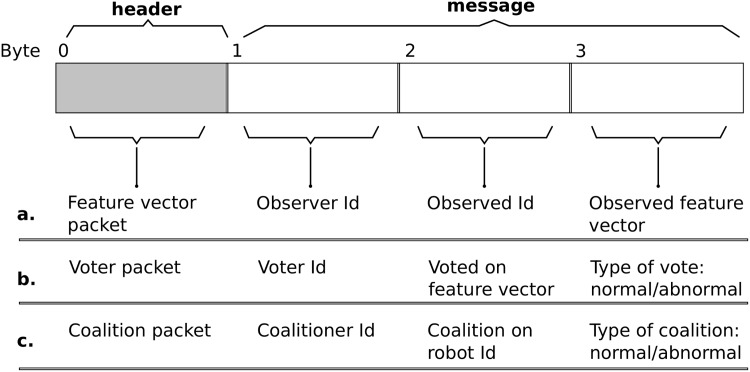
Types of data packets for inter-robot communication. A depiction of the data packet (4 bytes) transmitted by the range-and-bearing device of the robot. The first byte (shaded) identifies the header of feature vectors of observed robots, normal/abnormal votes on estimated feature vectors, and coalition information on observed robots. The remaining three bytes comprise the actual message, corresponding to each observed robot (feature vector and coalition packet types), and each observed feature vector (voter packet type).

6:  Communicate characterized feature vectors to neighboring robots, and similarly receive feature vectors estimated by neighbors (data-packet details, see Fig 2a).

7:  Delete feature vectors from observations older than *W*_*l*_ s.

8:  For each observed robot *r*_*j*_, improve the estimate on its feature vector *FV*_*j*_, using a simple majority vote on all received feature-vector information on *r*_*j*_.

9:  {Phase B: Classify behaviors of observed robots as normal/abnormal}

10:  Execute the CRM instance on all characterized feature vectors (Algorithm 3).

11:  **if** *time partitioned in W*_*l*_ *s* ≤ *W*_*l*_ − 1*s* **then**

12:   For each observed feature vector, update the number of times the observed feature vector is reported normal, and abnormal, by the CRM.

13:  **end if**

14:  {Phase C: Form inter-robot coalition on detected robot abnormalities}

15:  **if** *time partitioned in W*_*l*_ *s* > *W*_*l*_ − 1*s* **then**

16:   Receive coalition packet from neighbors (Fig 2c).

17:   Update local coalition list to include robots identified in received coalition packets.

18:   For each observed robot not on local coalition list, communicate vote on its behavior as normal/abnormal, based on simple majority on number of past instances it was detected normal/abnormal. Similarly, receive communicated votes from neighboring robots (Fig 2b).

19:   For each observed robot not in local coalition list, if number of received votes exceeds 5 (determined empirically), add robot to local coalition list.

20:   Send updated coalition list to neighbors.

21:  **end if**

22:  **if** *time is at end of partitioned W*_*l*_ *s time iterval* **then**

23:   Clear the local coalition list.

24:  **end if**

25: **end while**

To estimate the behavioral features, we define:

1. *R*: the swarm of robots, *r*_*i*_, *i* = 1…|*R*|, with 2*π* radians field of view of the onboard range-and-bearing sensors. Readings are noisy and have limited range of 1 m.
2. *O*_*i*_(*τ*): a set of observable robots *r*_*j*_ of *r*_*i*_, such that *r*_*j*_ ∈ *R* and *r*_*j*_ is located within sensing range of *r*_*i*_ at time *τ*.
  - Observed range *d*_*ij*_: ∀*r*_*j*_ ∈ *O*_*i*_(*τ*) the range of observed robot *r*_*j*_ from robot *r*_*i*_, with *d*_*ij*_ ∈ [0, 1] m.
  - Observed bearing *ϕ*_*ij*_: ∀*r*_*j*_ ∈ *O*_*i*_(*τ*) the bearing of observed robot *r*_*j*_ from robot *r*_*i*_, with *ϕ*_*ij*_ ∈ [0°, 360°].

#### 1. Features on observed robot’s immediate environment

The first two features $F1_{j}^{i}\left(\tau \right)$ and $F2_{j}^{i}\left(\tau \right)$ at time *τ* pertain to the number of neighbors of robot *r*_*j*_ as observed by *r*_*i*_.

For all the robots in the swarm *r*_*k*_ ∈ *O*_*i*_(*t*), the distance between the observed robot *r*_*j*_ and neighbors *r*_*k*_ (*j* ≠ *k*) can be estimated by robot *r*_*i*_ using the Law of Cosines as, $d_{jk}={\left(d_{ij}^{2}+d_{ik}^{2}-2d_{ij}d_{ik}\cos(\phi_{ij}-\phi_{ik})\right)}^{1/2}$.

Using the estimated distance *d*_*jk*_ at time *τ*, the neighbors *r*_*k*_ of the observed robot *r*_*j*_ (*k* ≠ *j*), in the inner range ([0, 15] cm), and the outer range ((15, 30] cm) are $Ni_{j}^{i}\left(\tau \right)\equiv \{r_{k}|r_{k}\in O_{i}\left(t\right)\wedge d_{kj}\in \left[0,15\right]\text{cm}\}$, and $No_{j}^{i}\left(\tau \right)\equiv \{r_{k}|r_{k}\in O_{i}\left(t\right)\wedge d_{kj}\in \left(15,30\right]\text{cm}\}$, respectively. Utilizing $Ni_{j}^{i}\left(\tau \right)$ and $No_{j}^{i}\left(\tau \right)$, the features $F1_{j}^{i}\left(\tau \right)$ and $F2_{j}^{i}\left(\tau \right)$ of robot *r*_*j*_ as observed by *r*_*i*_, are estimated as follows:

$$\begin{array}{c} F1_{j}^{i}\left(\tau \right)=1\;\text{if}\;\frac{\sum_{t=\tau }^{\tau -W_{l}}H[|Ni_{j}^{i}\left(\tau \right)\left|\right]}{W_{l}}>0.5\text{,}\;\text{otherwise}\;F1_{j}^{i}\left(\tau \right)=0 \end{array}$$ (1)

$$\begin{array}{c} F2_{j}^{i}\left(\tau \right)=1\;\text{if}\;\frac{\sum_{t=\tau }^{\tau -W_{l}}H[|No_{j}^{i}\left(\tau \right)\left|\right]}{W_{l}}>0.5\text{,}\;\text{otherwise}\;F2_{i}^{j}\left(\tau \right)=0 \end{array}$$ (2)

where $|Ni_{j}^{i}\left(\tau \right)|$ and $|No_{j}^{i}\left(\tau \right)|$ are the estimated number of neighbors of *r*_*j*_ as observed by *r*_*i*_ in the inner [0, 15] cm, and outer (15, 30] cm range, respectively, at time *τ*. Furthermore, *H*[.] is the Heaviside step function, defined as 1 if its argument exceeds 0, and 0 otherwise. At time *τ*, the features $F1_{j}^{i}\left(\tau \right)$ and $F2_{j}^{i}\left(\tau \right)$ are set, if the robot has at least one neighbor in range [0, 15] cm and (15, 30] cm, respectively, for the majority of the past observation time window *W*_*l*_ (parameters in Table 1).

#### 2. Features on observed robot’s action

The next two features $F3_{j}^{i}\left(\tau \right)$ and $F4_{j}^{i}\left(\tau \right)$, pertain to the motor actions of robot *r*_*j*_ observed by *r*_*i*_.

The feature $F3_{j}^{i}\left(\tau \right)$ encodes the distance traversed by the observed robot in the past observation time window *W*_*l*_. We here consider the forward kinematics of the robot, a non-holnomic differential-drive mobile robot. The robot’s local coordinate frame is placed at the center of the robot’s axle, and its y-axis aligned with the forward driving direction. In the local coordinate frame, the change in robot position and heading is estimated as follows (details in [25]):

$$\begin{array}{c} \Delta \omega_{i}=\frac{-v_{l}+v_{r}}{h}\Delta t \end{array}$$ (3)

$$\begin{array}{c} \Delta x_{i}=\frac{v_{l}+v_{r}}{2}\Delta t\cos\left(\omega_{i}+\Delta \omega_{i}/2\right) \end{array}$$ (4)

$$\begin{array}{c} \Delta y_{i}=\frac{v_{l}+v_{r}}{2}\Delta t\sin\left(\omega_{i}+\Delta \omega_{i}/2\right) \end{array}$$ (5)

where Δ*ω*_*i*_ and (Δ*x*_*i*_, Δ*y*_*i*_) is the change in heading *ω*_*i*_ and position (*x*_*i*_, *y*_*i*_), respectively, of robot *r*_*i*_ in the time interval Δ*t*. Additionally, *v*_*r*_ and *v*_*l*_ are the current linear speeds of the right and left wheels (in cm/s), respectively, of the robot with inter-wheel distance *h*.

**Algorithm 2 Subroutine to compute distance Dist[*r*_*i*_, *r*_*j*_, *W*] traversed by robot *r*_*j*_ over time window *W*, as estimated by observer robot *r*_*i*_.**

1: {Sense the range *d*_*ij*_ and bearing *ϕ*_*ij*_ of *r*_*j*_ with respect to *r*_*i*_}

2: {Estimate the initial position $\vec{P1_{i}^{j}}$ of robot *r*_*j*_ with respect to *r*_*i*_}

3: $\vec{P1_{i}^{j}}\leftarrow \left(d_{ij}\cos\left(\phi_{ij}\right),d_{ij}\sin\left(\phi_{ij}\right)\right)$

4: {Initialize heading and displacement vector of observer *r*_*i*_}

5: *ω*_*i*_ ← 0 and $\vec{v_{i}}\leftarrow \left(0,0\right)$

6: {Iterate over observation time window of *W* seconds}

7: **for** *t* = *current* to *current* + *W* **do**

8:  {Update the heading of the observer robot *r*_*i*_ (Eq (3))}

9:  *ω*_*i*_ ← *ω*_*i*_ + Δ*ω*_*i*_(*t*)

10:  {Update the displacement vector of the observer robot *r*_*i*_ (Eqs (4) and (5))}

11:  $\vec{v_{i}}\leftarrow \vec{v_{i}}+\left(\Delta x_{i}\left(t\right),\Delta y_{i}\left(t\right)\right)$

12: **end for**

13: {Sense the new range *d*_*ij*_ and bearing *ϕ*_*ij*_ of *r*_*j*_ with respect to *r*_*i*_}

14: {Estimate final position $\vec{P2_{i}^{j}}$ of robot *r*_*j*_ with respect to *r*_*i*_}

15: $\vec{P}\leftarrow \left(d_{ij}\cos\left(\phi_{ij}\right),d_{ij}\sin\left(\phi_{ij}\right)\right)$

16: {Transform estimated position to robot *r*_*i*_ local frame prior to elapsed *W*s}

17: $\vec{P2_{i}^{j}}\leftarrow \left(P_{x}\cos\left(\omega_{i}\right)-P_{y}\sin\left(\omega_{i}\right),P_{x}\sin\left(\omega_{i}\right)+P_{y}\cos\left(\omega_{i}\right)\right)+\vec{v_{i}}$

18: Return $\left|\right|\vec{P2_{i}^{j}}-\vec{P1_{i}^{j}}\left|\right|$

Using the relationship between the robot’s wheel speeds and the change in its position and heading (Eqs (3)–(5)), the distance traversed by *r*_*j*_ as observed by *r*_*i*_ is estimated with Algorithm 2. Subsequently, the feature $F3_{j}^{i}\left(\tau \right)$ of robot *r*_*j*_ as observed by *r*_*i*_, is estimated as follows:

The feature $F3_{j}^{i}\left(\tau \right)$ at time *τ* denotes if the robot *r*_*j*_, observed by *r*_*i*_ over observation time window *W*_*l*_, is moving. It is estimated as,

$$\begin{array}{cc} F3_{j}^{i}\left(\tau \right) & =1\;\text{if}\;\mathrm{Dist}\left[r_{i},r_{j},W_{l}\right]>0.15W_{l}\left|{\vec{v}}_{\text{max}}\right|\text{,}\;\text{otherwise}\;F3_{i}^{j}\left(\tau \right)=0 \end{array}$$ (6)

where the function Dist[*r*_*i*_, *r*_*j*_, *W*_*l*_] estimates the distance traversed by robot *r*_*j*_ as observed by *r*_*i*_ over time window *W*_*l*_, and with maximum linear speed $|{\vec{v}}_{\text{max}}|$. The feature $F3_{j}^{i}\left(\tau \right)$ is set at time *τ*, if the distance traversed exceeds 15% of the maximum distance that may be traversed by the robot in *W*_*l*_ s. The 15% threshold is set to compensate for odometric noise in estimating the observed robot position, from the range-and-bearing sensors and motor encoders of the observer robot.

The second motor-action feature $F4_{j}^{i}\left(\tau \right)$ encodes the proportion of observed instances the robot *r*_*j*_ alters its heading during motion, as observed by robot *r*_*i*_. For these interactions, a robot’s motor response corresponding to an alteration in heading during motion, is characterized as follows:

$$\begin{array}{c} M_{j}^{i}\left(\tau \right)=\left\{ \begin{array}{cc} 1, & \text{if}\;(\underset{\text{proprioception}}{\underset{︸}{\frac{{\ddot{\omega }}_{j}\left(\tau \right)}{{\ddot{\omega }}_{\text{max}}}}}\times \;\underset{\text{observation}}{\underset{︸}{\frac{\mathrm{Dist}[r_{i},r_{j},W_{s}]}{W_{s}\left|{\vec{v}}_{\text{max}}\right|}}})>0.1\\ 0, & \text{otherwise} \end{array}\right. \end{array}$$ (7)

where ${\ddot{\omega }}_{j}\left(\tau \right)/{\ddot{\omega }}_{\text{max}}$ is the normalized angular acceleration, proprioceptively computed by robot *r*_*j*_ and communicated to *r*_*i*_. The $\mathrm{Dist}\left[r_{i},r_{j},W_{s}\right]/(W_{s}|{\vec{v}}_{\text{max}}\left|\right)$ is the normalized distance traversed by *r*_*j*_ and observed by *r*_*i*_, over the short time window *W*_*s*_. This observed distance is used to robustly assess that the robot *r*_*j*_ is indeed moving, and not stuck (e.g., due to wheel slippage) while still registering a change in heading from its wheel encoders. A motor response for robot *r*_*j*_ is registered by *r*_*i*_, as $M_{j}^{i}\left(\tau \right)$, if the product of its normalized angular acceleration (proprioceptively computed by *r*_*j*_ and communicated to *r*_*i*_), and distance (observed by *r*_*i*_), exceeds 10% of maximum normalized value of 1. The 10% threshold is set to compensate for odometric noise in the proprioceptively computed angular acceleration, and in the estimated distance traversed by the observed robot.

The Bayes estimator is applied to robustly estimate the prevalence of instances an observed robot registers a motor response during its entire operation. Consider a robot *r*_*j*_ that during its entire operation has a fraction of control cycles *θ*_*j*_, in which it alters its heading during motion. The robot *r*_*i*_ with a limited sample of observations of *r*_*j*_, has to infer the posterior probability distribution of *θ*_*j*_.

Let the robot *r*_*i*_ have $D_{j}^{i}\left(\tau \right)$ observations of *r*_*j*_ at time *τ*, $X_{j}^{i}\left(\tau \right)$ of which involve an observed change in the robot’s heading:

$$\begin{array}{ccc} D_{j}^{i}\left(\tau \right) & \equiv & \left\{M_{j}^{i}\left(t\right)|t\in \left\{0\ldots \tau \right\}\wedge r_{j}\left(t\right)\in O_{i}\left(t\right)\right\}\\ X_{j}^{i}\left(\tau \right) & \equiv & \left\{M_{j}^{i}\left(t\right)|t\in \left\{0\ldots \tau \right\}\wedge r_{j}\left(t\right)\in O_{i}\left(t\right)\wedge M_{j}^{i}\left(t\right)=1\right\} \end{array}$$ (8)

The likelihood function *P*(*D*|*θ*), i.e. the probability model believed to have generated the observations *D*(*τ*), can be defined as a binomial distribution (|D||X|)θ|X|(1-θ)|D|-|X| (robot and time index notations removed for brevity). The prior probability distribution $P(\theta_{j}^{i})$ is robot *r*_*i*_’s a priori belief on the instances the observed robot *r*_*j*_ changes its heading during operation. With no information on *θ* it can be defined as a uniform distribution, specified as beta distribution Beta(*α*_0_, *β*_0_), with *α*_0_ and *β*_0_ initialized to 1.

Bayes’ theorem states,

$$\begin{array}{c} P\left(\theta_{j}^{i}\left(\tau \right)|D_{j}^{i}\left(0:\tau \right)\right)\propto P\left(\theta_{j}^{i}\left(\tau \right)|D_{j}^{i}\left(0:\tau -1\right)\right)P\left(D_{j}^{i}\left(\tau \right)|\theta_{j}^{i}\left(\tau \right)\right) \end{array}$$ (9)

where $P(\theta_{j}^{i}\left(\tau \right)|D_{j}^{i}\left(0:\tau \right))$ is the posterior probability distribution after taking observations into account.

Since the prior is defined as a beta distribution, and we apply a binomial likelihood function, the posterior is derived to be a beta distribution (for details see conjugate priors of exponential family [26]), specified as follows:

$$\begin{array}{c} P\left(\theta_{j}^{i}\left(\tau \right)|D_{j}^{i}\left(0:\tau \right)\right)=\mathrm{Beta}(\alpha_{o}+|X_{j}^{i}\left(\tau \right)|,\beta_{0}+|D_{j}^{i}\left(\tau \right)|-|X_{j}^{i}\left(\tau \right)\left|\right) \end{array}$$ (10)

Using the closed-form expression of the posterior distribution (Eq (10)) to compute its expected value, the feature $F4_{j}^{i}\left(\tau \right)$ of robot *r*_*j*_ as observed by *r*_*i*_ is set if the expected proportion of instances the observed robot *r*_*j*_ alters its heading exceeds a threshold of 5% to compensate for stochastic variation in robot behavior.

$$\begin{array}{c} F4_{j}^{i}\left(\tau \right)=1\;\text{if}\;E\left(\theta_{i}^{j}\left(\tau \right)|D_{j}^{i}\left(0:\tau \right)\right)>0.05\text{,}\;\text{otherwise}\;F4_{i}^{j}\left(\tau \right)=0 \end{array}$$ (11)

where $E(\theta_{i}^{j}\left(\tau \right)|D_{j}^{i}\left(0:\tau \right))$ is the expected value of the posterior distribution at time *τ*, defined for Beta(*α*, *β*) as *α*/(*α* + *β*).

#### 3. Features on observed robot’s sensorimotor interactions

The final two features, $F5_{i}^{j}\left(\tau \right)$ and $F6_{i}^{j}\left(\tau \right)$, pertain to the robot *r*_*j*_ sensorimotor interactions, as observed by *r*_*i*_. For these interactions, we define two sensorimotor interaction events Sm and Sn as follows:

$$\begin{array}{c} Sm_{j}^{i}\left(\tau \right)=\;H[|Ni_{j}^{i}\left(\tau \right)\cup No_{j}^{i}\left(\tau \right)\left|\right]\wedge M_{j}^{i}\left(\tau \right) \end{array}$$ (12)

$$\begin{array}{c} Sn_{j}^{i}\left(\tau \right)=\neg H[|Ni_{j}^{i}\left(\tau \right)\cup No_{j}^{i}\left(\tau \right)\left|\right]\wedge M_{j}^{i}\left(\tau \right) \end{array}$$ (13)

The above sensorimotor interaction event $Sm_{j}^{i}\left(\tau \right)$ at time *τ* is set if observed robot *r*_*j*_ alters its heading in the presence of sensory input (one or more neighbors in range), and as observed by *r*_*i*_. Similarly, $Sn_{j}^{i}\left(\tau \right)$ is set if *r*_*j*_ alters its heading in the absence of sensory input (no neighbors in range).

Bayesian inference is applied to estimate the features F5 and F6, using the binomial likelihood function and the beta prior and posterior probability distribution specifications. The observations driving the posterior distributions (P(θm|Dm) for F5, and P(θn|Dn) for F6) are defined as follows:

$$\begin{array}{ccc} Dm_{j}^{i}\left(\tau \right) & \equiv & \left\{Sm_{j}^{i}\left(t\right)|t\in \left\{0\ldots \tau \right\}\wedge r_{j}\left(t\right)\in O_{i}\left(t\right)\right\}\\ Xm_{j}^{i}\left(\tau \right) & \equiv & \left\{Sm_{j}^{i}\left(t\right)|t\in \left\{0\ldots \tau \right\}\wedge r_{j}\left(t\right)\in O_{i}\left(t\right)\wedge Sm_{j}^{i}\left(t\right)=1\right\} \end{array}$$ (14)

$$\begin{array}{ccc} Dn_{j}^{i}\left(\tau \right) & \equiv & \left\{Sn_{j}^{i}\left(t\right)|t\in \left\{0\ldots \tau \right\}\wedge r_{j}\left(t\right)\in O_{i}\left(t\right)\right\}\\ Xn_{j}^{i}\left(\tau \right) & \equiv & \left\{Sn_{j}^{i}\left(t\right)|t\in \left\{0\ldots \tau \right\}\wedge r_{j}\left(t\right)\in O_{i}\left(t\right)\wedge Sn_{j}^{i}\left(t\right)=1\right\} \end{array}$$ (15)

where at time *τ*, $Dm_{j}^{i}\left(\tau \right)$ and $Dn_{j}^{i}\left(\tau \right)$ comprise the sample of past observations of robot *r*_*j*_ as observed by *r*_*i*_, with and without neighbors in range, respectively. Similarly, $Xm_{j}^{i}\left(\tau \right)$ and $Xn_{j}^{i}\left(\tau \right)$ comprise the sample of past observations of *r*_*j*_ made by *r*_*i*_ altering its heading, with and without any neighbors in range, respectively.

Using the data of past observations, and the binomial likelihood function, the posterior distribution is specified as follows:

$$\begin{array}{c} P\left(\theta m_{j}^{i}\left(\tau \right)|Dm_{j}^{i}\left(0:\tau \right)\right)=\mathrm{Beta}(\alpha_{o}+|Xm_{j}^{i}\left(\tau \right)|,\beta_{0}+|Dm_{j}^{i}\left(\tau \right)|-|Xm_{j}^{i}\left(\tau \right)\left|\right) \end{array}$$ (16)

$$\begin{array}{c} P\left(\theta n_{j}^{i}\left(\tau \right)|Dn_{j}^{i}\left(0:\tau \right)\right)=\mathrm{Beta}(\alpha_{o}+|Xn_{j}^{i}\left(\tau \right)|,\beta_{0}+|Dn_{j}^{i}\left(\tau \right)|-|Xn_{j}^{i}\left(\tau \right)\left|\right) \end{array}$$ (17)

where *α*_*o*_ and *β*_*o*_ are initialized to 1, specify the prior probability distribution Beta(*α*_0_, *β*_0_) as a uniform distribution.

Utilizing Eqs (16) and (17), the features $F5_{j}^{i}\left(\tau \right)$ and $F6_{j}^{i}\left(\tau \right)$ of robot *r*_*j*_ as observed by *r*_*i*_, is estimated as follows:

$$\begin{array}{c} F5_{j}^{i}\left(\tau \right)=1\;\text{if}\;E\left(\theta m_{i}^{j}|Dm_{j}^{i}\left(0:\tau \right)\right)>0.05\text{,}\;\text{otherwise}\;F5_{i}^{j}\left(\tau \right)=0 \end{array}$$ (18)

$$\begin{array}{c} F6_{j}^{i}\left(\tau \right)=1\;\text{if}\;E\left(\theta n_{i}^{j}|Dn_{j}^{i}\left(0:\tau \right)\right)>0.05\text{,}\;\text{otherwise}\;F6_{i}^{j}\left(\tau \right)=0 \end{array}$$ (19)

where the features $F5_{j}^{i}\left(\tau \right)$ and $F6_{j}^{i}\left(\tau \right)$ are set at time *τ*, if the observed robot *r*_*j*_ alters its heading at least 5% of the time, in the presence, and in the absence of sensory input, respectively. For both the features, the 5% threshold is set to compensate for stochastic variation in robot behavior.

#### Voting to improve estimation of behavioral features

We take advantage of the multiplicity of robots in large-scale robot swarms to increase the robustness of the estimation of behavioral features. Robots share behavioral features observed up to *W*_*l*_ s in the past with their neighbors. A voting scheme is then employed by each robot to select the most likely observed feature value, for each of the six features.

At each simulated time-instance *τ*, every robot *r*_*i*_ of the swarm *R* observes its neighbors *r*_*j*_ ∈ *O*_*i*_(*τ*), to estimate its behavioral feature vector $\left(F1_{i}^{j}\left(\tau \right),F2_{i}^{j}\left(\tau \right),\ldots ,F6_{i}^{j}\left(\tau \right)\right)$ using Eqs (1)–(19). Subsequently, *r*_*i*_ communicates to all neighboring robots *O*_*i*_(*τ*), voter packets comprising the observed robot identifications (in range [1, |*R*|]) and their corresponding estimated feature vectors (data-packet structure, see Fig 2a). The final feature vector for each observed robot is then updated by a simple majority vote on the most recently received feature-vector information in the past *W*_*l*_ s, counting each voter only once. In considering voter information up to *W*_*l*_ s in the past, we assume that the robot behavior does not change within this time.

### B. Detection of faulty robots

In the next phase of fault detection (Algorithm 1 Phase B), an instance of the CRM is run on each robot to classify the observed behavioral feature vectors as normal/abnormal. The behaviors in the swarm that are persistent and abundant (performed by most of robots) are to be treated as normal. By contrast, rare behaviors (exhibited by fewer robot) are to be classified as abnormal. Below, we outline the CRM and describe its functioning, that are later embodied distributedly in a multirobot system.

**Algorithm 3 Subroutine to classify behavioral feature vectors of observed robots as normal/abnormal (simulation of a CRM instance).**

1: Compute the distribution of estimated feature vectors *FV*_*j*_ of the observed robots.

2: Assign feature vectors to APCs i.e., ∀*j*, *A*_*j*_ = *FV*_*j*_.

3: ∀*j* ∈ {1, 2…*M*}, if *A*_*j*_ > 0, increment $T{\;E}_{j}$ and $T{\;R}_{j}$ by IE and IR, respectively.

4: **while** *time* ≤ *S* **do**

5:  ∀*i* ∈ {1, 2…*M*} and *T*_*i*_ > 0, and ∀*j* ∈ {1, 2…*M*} where *A*_*j*_ > 0, compute the number of conjugated cells *C*_*ij*_ in quasi-steady state.

6:  Using the number of conjugated cells, compute the updated number of effector and regulatory cells with the Euler-Heun adaptive step method [27].

7:  Increment *time*

8: **end while**

9: For each feature vector, compute the sum of effector and regulatory cells, weighted by their affinity.

10: Tolerate the feature vector as normal if total regulatory cells exceeds effector cells, else interpret it as abnormal.

#### Crossregulation model

The CRM describes the population dynamics of cells of the adaptive immune system, consisting of three mutually interacting cell types: (i) antigen presenting cells (APCs) that present the antigen on their surface. Individual APCs have a fixed number of conjugation sites (*s*) on which effector and regulatory T-cells can form conjugates; (ii) effector cells T_E_ that can potentially mount immune responses which, depending on receptor specificity, may be directed to foreign pathogens or to body-antigens; and (iii) regulatory cells T_R_ that suppress proliferation of T_E_ cells with similar specificities. Furthermore, APCs are classified into different sub-populations of equivalent APCs, with each APC in a sub-population presenting the same antigen on its surface. Similarly, effector and regulatory cells are also classified into different clones according to their specificity.

A mathematical formulation using ordinary differential equations, of the dynamics of interactions between effector and regulatory cells, with APCs, is detailed in [19]. In this section, we describe these inter-cellular interactions, introduce the important parameters, followed by the algorithmic implementation of the model for a multirobot system.

#### Functioning of the model

The CRM implemented on each robot of the multirobot system is as follows. The CRM provides a differential equation governing each of the clonal types (*i*) of effector ($TE_{i}$), and regulatory ($TR_{i}$) T-cells. The sub-populations of each of these clonal types is subject to the following: (i) a constant influx of new cells; (ii) growth by proliferation (division of parent cells to two daughter cells) of their individual activated cells; and (iii) shrinkage consequent to death of T-cells (see Table 2 for influx, proliferation and death rates of TE and TR cells). All T-cell clones are generated with similar initial conditions i.e., ∀*i*, $T{\;E}_{i}\left(0\right)=T{\;E}_{0}$ and $T{\;R}_{i}\left(0\right)=T{\;R}_{0}$.

**Table 2:** Parameters of the crossregulation model.

Param.	Description	Value (a.u.)
*A*_*j*_	Density of APCs of sub-population *j*	–
*s*	Maximum number of T-cells that can bind to an APC	3
$T{\;E}_{0}$	Seed density of effector cells	10
$T{\;R}_{0}$	Seed density of regulatory cells	10
$T{\;E}_{i}$	Density of effector cells of clone *i*	–
$T{\;R}_{i}$	Density of regulatory cells of clone *i*	–
*T*_*i*_	Density of T-cells of clone *i*	$T{\;E}_{i}+T{\;R}_{i}$
*C*_*ij*_	Density of conjugates between *T*_*i*_ and *A*_*j*_	–
*γ*	Conjugation and deconjugation rates of T-cells and APCs	10^−1^
*π*_*E*_	Proliferation rate of effector cells	10^−3^
*π*_*R*_	Proliferation rate of regulatory cells	0.7 × 10^−3^
*δ*	Death rate of effector and regulatory cells	10^−6^
*M*	Maximum number of different 6-bit feature vectors	2^6^
*c*	Cross-reactivity between T-cells and APCs	0.15
IE	Density of new effector cells introduced at each control-cycle	10
IR	Density of new regulatory cells introduced at each control-cycle	10
*S*	Time CRM instance is executed, in a single control-cycle	5 × 10^7^

The density of activated $TE_{i}$ and $TR_{i}$ cells of each clonal type *i*, is dependent on their interactions with APCs *A*_*j*_ of each sub-populations *j*. For example, let us consider the interactions between the *i*-th T-cell clone and the *j*-th APC population. The resulting conjugates *C*_*ij*_ is subject to the following: (a) formation of new conjugates by the free T-cells of clone *i* with available conjugation sites on APCs of sub-population *j*. This conjugation rate is also controlled by the affinity between the T-cells clone *i* and APCs sub-population *j*; and (b) dissociation of existing conjugated T-cells from APCs (see Table 2 for the conjugation and deconjugation rates, and the affinity between T-cells and APCs). The conjugation and deconjugation of T-cells from APCs are fast processes with respect to the overall T-cell clone dynamics. Consequently, we solve analytically at each time step, the quasi-steady state values of the conjugates. Finally, the density of activated effector and regulatory cells is computed from the quasi-steady state densities of the conjugates, utilizing the Euler-Heun adaptive step method [27]. The conjugated effector cells are activated in the absence of regulatory cells on the same APC. In contrast, conjugated regulatory cells can only be activated if at least one effector cell is simultaneously conjugated to the same APC.

In the CRM, at low concentrations of APCs, the system always reaches a globally stable state consisting solely of TE cells (immune response). By contrast, at higher concentrations of APCs, the system exhibits bistable behavior, i.e., the system can evolve either into an equilibrium state composed predominantly of TE cells (immune response), or into a state consisting mostly of TR cells (tolerant response). The system evolves into the TR cell dominated state, provided that the seeding population has sufficient TR cells. The seed T-cell population density ($T{\;E}_{0}$ and $T{\;R}_{0}$) is chosen to ensure a tolerant response in the bistable parameter regime.

#### CRM in a multirobot system

In this section, we demonstrate how the CRM is implemented on a distributed embodied multirobot system in order to give the system the capacity to detect abnormally behaving robots, while maintaining a tolerance towards normal swarm behavior.

In the model’s implementation, at each control cycle, every robot of the swarm computes the distribution of estimated feature vectors: the number of its observed neighbors associated with each its estimated feature vectors (*FV*_*j*_). In the robot’s internal CRM instance, APCs are then generated corresponding to each of the feature vectors perceived. Each APC presents an individual feature vector to the T-cells. The number of each type of the APCs generated *A*_*j*_ = *FV*_*j*_, for *j* ∈ {1, …, *M*}, where *M* = 2^6^ is the maximum number of different 6-bit feature vectors perceived by the robot.

The T-cell clones (*T*_1_, *T*_2_, …, *T*_*M*_), each have a different receptor encoded as a binary string, which determines their affinity to the APC population. The affinity between T-cell clonal *i* and APC population *j* is denoted by *θ*_*ij*_:

$$\begin{array}{c} \theta_{ij}=\exp(-\frac{D_{H}\left[i,j\right]}{c}) \end{array}$$ (20)

where $D_{H}$ is the Hamming distance between the receptor of *T*_*i*_ and the feature vector presented by *A*_*j*_, and *c* is the cross-reactivity between T-cells and APCs. The affinity *θ*_*ij*_ decreases with an increase in the dissimilarity between the T-cell receptor *T*_*i*_ and the feature vector presented by *A*_*j*_. The sharpness of this decrease in affinity is modulated by the cross-reactivity *c* of the system. For instance, a high value of *c* would result in all T-cell clones having a high affinity to all APC populations. By contrast, at low *c*, each T-cell clone *T*_*i*_ would have a high affinity to only one distinct APC population *A*_*j*_, for which *D*_*H*_[*i*, *j*] = 0 (for details on modeling the cross-reactive immune response, see [28, 29]).

At the start of the simulation, the number of effector and regulator cells on each robot is initialized to $T{\;E}_{0}$ and $T{\;R}_{0}$ respectively. Following this, Algorithm 3 (parameters in Table 2) is executed by the robots in each control cycle, allowing the robots to run their internal CRM instance. The robots begin by computing the distribution of estimated feature vectors, and the corresponding density of APCs. The CRM is then numerically integrated for time *S*, allowing the system to respond to the different APCs. Finally, the robot decides the nature of each observed feature vector *FV*_*j*_ by first computing the following quantities:

$$\begin{array}{ccc} T\;E & = & \sum_{i=1}^{M}\theta_{ij}T{\;E}_{i}\\ T\;R & = & \sum_{i=1}^{M}\theta_{ij}T{\;R}_{i} \end{array}$$ (21)

and tolerating the feature vector as normal if TR>TE. By contrast, if TE>TR, the feature vector is classified as abnormal.

#### Detection of abnormally behaving robots

A reliable detection of abnormally behaving robots is obtained by accumulating the CRM output over a series of consecutive control cycles instead of classifying the behavioral feature vectors based on the CRM output in a single control cycle. Depending on the relative cost of tolerating an abnormally behaving robot (false negatives) and of incorrectly classifying a robot as behaving abnormally (false positives), the output may be interpreted in one of several ways. A simple scheme involves storing the past *n* outputs of the CRM-based abnormality detector and only detecting a robot as abnormal if its feature vector has been classified as such for the majority of past *n* control cycles. If fault accommodation is expensive, while the presence of abnormally behaving robots has a relatively small impact on performance, *n* could be set to a relatively high value. Conversely, in critical tasks where abnormal behavior can be catastrophic, *n* could be set to a relatively low value.

In our implementation, *n* is set to 90 (corresponding to 9 s), to provide a suitable trade-off between the latency in the detection of the faulty robot, and the number of false-positive incidents. The robot is detected as behaving abnormally iff its feature vector has been classified as such for the majority of the past *n* control-cycles. Otherwise, the robot is treated as behaving normally.

### C. Distributed coalition formation on detected abnormal behavior

In order to accommodate an abnormally behaving or faulty robot, the robots of the swarm have to first consolidate their individual decisions on their neighboring robots based on their respective CRM instances, into a swarm-level decision on the normal/abnormal state of the robots. Such a swarm-level agreement on the state of individual robots is formed via coalition formation process, implemented distributedly in a multirobot system, and described below.

At the end of the 9 s interval, individual robots of the swarm accumulate their CRM output to decide on the normal/abnormal status of their neighboring robots. Following this, Algorithm 1 (Phase C) is executed to form coalitions on the status of individual robots, within the duration of 1 s. All the robots of the swarm maintain an individual local coalition list of the identification *i* of the robots on which coalition has been formed (*i* ∈ [1, |*R*|]), and the result of the coalition (swarm’s decision on normal/abnormal status of *i*). During the 1 s interval allocated to form coalitions, each robot votes (as normal/abnormal) on the behavioral feature vectors of neighboring robots on which a coalition has not yet been formed, by communicating a voter data packet (Fig 2b). The neighboring robots accumulate the votes received on the feature vectors, counting each voter only once. Robots that receive more than five votes (determined empirically, aiming to reduce false positive rate) on a behavioral feature vector add the corresponding robot to their local coalition list, along with the results of the vote. The updated local coalition list is subsequently propagated across the swarm, using coalition data packets (Fig 2c). Finally, at the end of the allocated 1 s interval, every robot of the swarm clears its local coalition lists, to allow new coalitions to be formed in response to changes in robot behavior.

## Experimental setup

We use a physics-based, discrete-time multirobot simulator named ARGoS [30], designed to realistically simulate complex experiments involving large swarms of robots. We simulate a robot swarm composed of 20 e-puck robots [24] situated in an environment with a size of 3 × 3 m^2^. The e-puck robot has a diameter of 7.5 cm, a maximum speed of 10 cm/s, and a control cycle of 0.1 s (full list of robot parameters in Table 1). In our experiments, the e-puck robot model is equipped with eight infrared proximity sensors for obstacle avoidance and two actuators which control the robots movement speed and direction. Each robot is also equipped with a range and bearing extension board [31], which enables a robot to estimate the relative location and orientation of neighboring robots.

A faithful simulation of the physical e-puck is accomplished by adding noise to the values of the sensor readings, and to the desired actuator speeds of the simulated robot, so as to simulate the stochasticity inherently associated with real robots (detailed description of employed noise model in [32]). Infrared proximity sensors on the robot have a limited range of 10 cm, register a reading between [0, 1], and is subject to additive uniform noise within ±0.1. By contrast, the range-and-bearing sensors of the robot have a higher range of up to 100 cm, and are simulated with the addition of a noise vector to the sensor reading. The length of the added vector follows a Gaussian distribution N[0 *cm*, 1 *cm*], and the angle of the vector is simulated with uniform noise within ±*π* radians. The simulated robots motor encoders employ an additive uniform noise within ±0.1 cm/s, applied on the readings of motor speed of the left and right wheels of the robot. Finally, the motors driving the two wheels of the robot are simulated with a multiplicative noise model following a Gaussian distribution N[0, 0.1], and applied independently on the desired left and right wheel speeds of the robot.

### Swarm behaviors

In our fault-detection system, the behaviors of the robots in a swarm are classified as normal/abnormal, where abnormalities are consequent to faults in the robot. For our experiments, fault detection is evaluated for both homogeneous and heterogeneous normal swarm behaviors.

### Homogeneous swarm behaviors

In the homogeneous swarm behaviors, all the robots of the swarm execute an identical swarm behavior during the entire duration of the simulation. The swarm behaviors simulated are (i) dispersion, (ii) aggregation, (iii) flocking, and (iv) homing towards a stationary landmark. At the start of the experiment, the robots are randomly placed in the 3 × 3 m^2^ environment. The robot behaviors are implemented using a subsumption architecture [33]. In dispersion, the robots move in the opposite direction of the center of mass of their neighbors. By contrast, in aggregation, the robots move towards the center of mass of surrounding neighbors, but disperse away if too close to their neighbors to avoid collisions. Similarly, homing robots move towards a single pre-specified stationary beacon that serves as a landmark, and move away if too close to the landmark or to other robots. The position of the beacon for homing is selected at random at the start of the experiment. Finally, in flocking, the robots continually adjust their velocity to that of neighboring robots, where the velocity of the neighboring robots is estimated over a time-window of *W*_*s*_ s. The flocking robots aggregate towards and disperse from neighbors, if they are too far away or close by, respectively (for source code details see Section A in S1 File).

### Heterogeneous swarm behaviors

**Fig 3:**
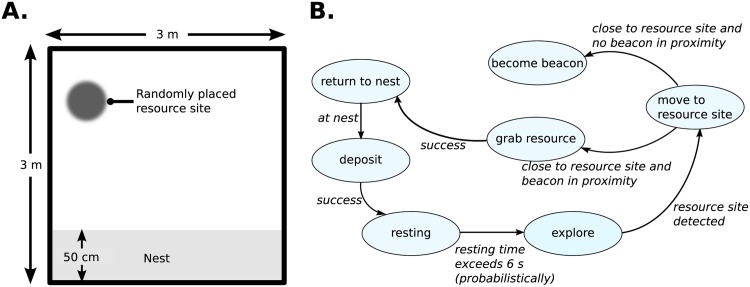
Foraging experiment setup for heterogeneously behaving swarm of 20-e-puck robots. A: The 9 m^2^ arena comprising a nest site, and the foraging space with a randomly placed resource site. B: Finite state machine diagram controlling each and every robot of the swarm.

The heterogeneous swarm behaviors require the robots of the swarm to execute complex (or composite) behaviors, thus entailing the robots of the swarm to often exhibit distinct behaviors at any given time of the simulation. In swarm robotics, foraging serves as an metaphor for a broad range of important problems involving: cooperative exploration, navigation, resource localisation, and recruitment [34, 35]. Therefore, in our experiments, the heterogeneous swarm behavior simulated is cooperative foraging, wherein the robots of the swarm have to locate a randomly placed resource site, gather resources from this site, and return the foraged resources to a designated nest area (Fig 3a). For successful foraging, the following repertoire of different behaviors are exhibited by individual robots of the swarm: (i) searching for resource site; (ii) signaling the presence of new resource; (iii) homing towards signaled resource site; and (iv) collecting and returning foraged resource to the nest. A finite state machine architecture is employed to control the robot foraging behavior (see Fig 3b). At the start of the experiment, the robot swarm is positioned at a nest site located at one end of the foraging arena. The robots begin foraging by exploring their environment, employing the dispersion swarm behavior. Upon locating the resource site, and with no existing beacon robots in its vicinity, the discoverer robot transitions into a beacon. The beacon robot transmits a foraging signal utilizing its range-and-bearing sensors. Exploring robot in range of the beacon home towards the foraging signal, and upon locating the resource site, simulate the gathering of resources by remaining stationary at the resource site. Once the resources are gathered, the foraging robot returns to the nest site. At the nest, robots simulate the depositing of resources by remaining stationary at the nest site. Upon successfully depositing the foraged resources at the nest, the robots return back to the exploration state.

### Faulty behaviors

In our multirobot simulator, faults are simulated directly in the robots sensors and actuators. Consequently, the resulting faulty behavior of the robot correspond to the actions performed by its controller, that is either provided with input from faulty sensors, or whose commands to actuators are not executed correctly by the underlying hardware. The faults simulated in our experiments are representative of seven different scenarios involving permanent failure in the e-puck robots sensor and actuator devices (fault types detailed in Table 3).

**Table 3:** Types of faults, simulated on one robot, selected at random from the swarm.

Fault type	Description	Fault simulation†	Devices affected
Fixed value sensor faults	One or few sensor fail permanently and return a fixed value	The faulty sensors return either, the smallest possible sensory value of 0 (**PMIN**), the largest possible sensory value of 1 (**PMAX**), or a randomly selected value U[0, 1] for each control-cycle the fault occurs (**PRND**).	Four frontal IR proximity sensors of the e-puck.
Sensor offset faults	One or few sensor fail permanently and return a reading offset by a random value	The faulty sensors returns the true reading (undamaged sensor reading) altered by an additional value, randomly selected U[75 *cm*, 100 *cm*] for range and U[−*π*, *π*] on bearing for each control-cycle the fault occurs (**ROFS**).	Range and bearing sensors of the e-puck.
Motor faults	The motors of the robot malfunction, thus preventing the robot from rotating the affected wheel(s).	The desired wheel speed corresponding to the faulty motor is set to 0 cm/s when the fault occurs (left wheel **LACT**, right wheel **RACT**, both wheels **BACT**); subsequent commands to change the wheel speed are ignored.	Left, right, or both the motors of the e-puck.

† Function U[*a*, *b*] returns a randomly selected value from a uniform distribution between *a* and *b*.

#### Infrared proximity sensors

For the infrared proximity sensors, the faults represent scenarios involving disconnected proximity sensors (fault type **PMIN**), obstructions (e.g., a piece of dust) stuck on the proximity sensors (fault type **PMAX**), and scenarios in-between these two extremes such as an obstruction on the proximity sensor is temporarily and partially dislodged as the robot moved about the environment (fault type **PRND**).

#### Range-and-bearing sensors

The fault on the range-and-bearing sensors (fault type **PRAB**) assumes that all the range-and-bearing receivers of the robot are identically affected by the fault. Consequent to this fault, the strength of the received signal on the range-and-bearing receivers is impeded so that objects that are near to the faulty robot appear to be far.

#### Actuators

The faults in the e-pucks motors occur when one or both motors of the robot malfunction, or if the tire on the robot’s wheel rim wears out. This fault prevents the robot from rotating the affected wheel (left wheel **LACT**, right wheel **RACT**, both wheels **BACT**).

## Results

In this section, we assess the performance of our fault-detection algorithm in different scenarios. We first evaluate the capacity of a multirobot system to detect robots behaving faulty for different combinations of normal (homogeneous and heterogeneous) behaviors, and faulty behaviors. We then evaluate how frequently normally behaving robots are misclassified as behaving faulty, and the influence of transitions in normal behavior on performance. Finally, we assess the importance of the different components of our algorithm for successful fault detection.

### Robot swarm detects almost all injected faults

**Fig 4:**
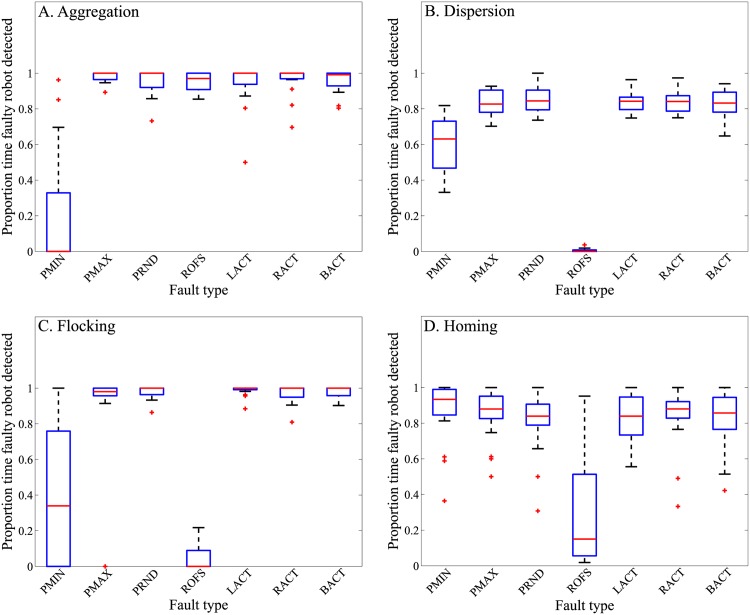
Fault detection for homogeneously behaving robot swarm. Proportion of time the faulty robot is detected across 20 replicates, in each of the 28 distinct combinations of normal (aggregation, dispersion, flocking and homing) homogeneous swarm behaviors, and fault types (PMIN, PMAX, PRND, ROFS, LACT, RACT, and BACT). On each box, the mid-line marks the median, and the box extends from the lower to upper quartile below and above the median. Whisker outside the box indicate the maximum and minimum values, except in case of outliers, which are shown as crosses. Outliers are data points outside of 1.5 times the interquartile range.

**Fig 5:**
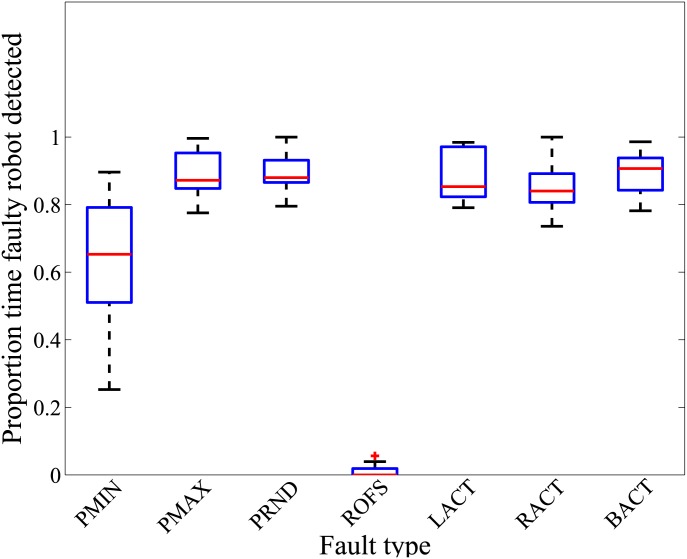
Fault detection for heterogeneously behaving robot swarm. Proportion of time the faulty robot is detected across 20 replicates, in each of the seven distinct combinations of normal (cooperative foraging) swarm behavior, and fault types (PMIN, PMAX, PRND, ROFS, LACT, RACT, and BACT).

We ran experiments with a swarm of 20 e-puck robots. In the swarm, 19 of the 20 robots performed one of the normal behaviors, that is, the aggregation, dispersion, flocking, or homing homogeneous behaviors, or the foraging heterogeneous behavior. The remaining one robot performed one of the faulty behaviors, PMIN, PMAX, PRND, ROFS, LACT, RACT, and BACT. We ran 20 replicates for each of the 35 combinations of 5 normal and 7 faulty behaviors. Each replicate lasted 6,000 cycles (corresponding to 600 s), and we recorded the proportion of time during which the swarm formed a majority coalition identifying the faulty robot. The results are presented in the box-plots in Fig 4 for homogeneous swarm behaviors, and Fig 5 for heterogeneous swarm behaviors, with one box for each combination of normal and faulty behavior.

#### Detection of faults injected in *homogeneously* behaving swarms

The results show that the median proportion of the time that the faulty robot was detected is above 0.50 in 23 out of the 28 experimental setups with homogeneous swarm behaviors (see Fig 4). The multirobot system successfully detected the faulty robot exhibiting fault types PMAX, PRND, LACT, BACT, and RACT, achieving a mean performance of 0.89 ± 0.14 (across the four homogeneous swarm behaviors). By contrast, the robots exhibiting fault types PMIN and ROFS were only detected in 0.55 ± 0.36 and 0.32 ± 0.41 proportion of time, respectively.

The poor fault-detection performance of fault types PMIN and ROFS, for the normal homogeneous swarm behaviors of aggregation, dispersion, flocking and homing, is ascribed to the following: (i) the malfunctioning sensor/motor device is unused in the experimental setup, and thus has not effect on the faulty robots behavior (condition termed Unused-Device); (ii) the normal behaving robots of the swarm compensate for the faulty robots behavior, thus preventing a disruption of the swarms behavior (Swarm-Compensate); and (iii) the initial random placement and orientations of the robots in some experiment replicates, and the subsequent interactions between normally behaving robots and the faulty robot, results in their behaviors being indistinguishable (Initial-Position).

In Table 4, we explain the situations that caused the poor fault detection performance, in the context of specific normal swarm behaviors.

**Table 4:** Experiment conditions resulting in false-negative incidents in the detection of faulty robots.

Unused-Device
*ROFS/dispersion:* The dispersing robots do not using the range-and-bearing sensor. Consequently, their behavior is unaffected by the malfunctioning sensor.
Swarm-Compensate
*PMIN/aggregation, flocking:* The robot with malfunctioning proximity sensors moves close in proximity to its neighbors using its range-and-bearing sensors, thus forming part of the robot aggregate. In the aggregate, the neighboring robots move out of the way of the faulty robot, thus compensating for its abnormal behavior, and preventing collisions between the robots. Similarly for flocking, as the range-and-bearing sensor is used to join a flock, and once in a flock the neighboring robots move out of the way to avoid collisions, flocking continues unhindered. When the abnormally behaving robot suffering from PMIN is temporarily stuck moving against a wall, out of range of the aggregate, it is detected as faulty.
*ROFS/flocking:* For faults in robots manifested by introducing an offset in the range-and-bearing sensor reading (selected at random at the start of each control cycle), due to the large size of the flocking swarm, as long as the faulty robot heads in the general direction of the flock (control cycles where a small bearing offset is introduced), it continues to follow in close proximity (approximately 15 cm from nearest neighbour in flock).
Initial-Position
*ROFS/homing:* In the replicates when the homing beacon is positioned close to the arena wall, instead of having all the robots of the swarm homing around the beacon, we have some dispersing robots continually moving in and out of range of the beacon. The robot inflicted with the fault type ROFS behaves similarly to these dispersing robots, and is therefore classified as normal by the swarm.

#### Detection of faults injected in *heterogeneously* behaving swarms

The performance of our fault-detection algorithm was not affected by the robot swarms performing composite swarm behaviors (Fig 5). During cooperative foraging, the robots of the swarm spent 64%, 6%, 2%, and 28% of their time assigned to exploration, signaling, retrieval of resources at foraging site, and returning foraged resource to the nest, respectively (mean across 20 robots, and 6,000 control-cycles). Despite the complexity of the task, the multirobot system successfully detected the faulty robots exhibiting fault types PMIN, PMAX, PRND, LACT, BACT, and RACT, achieving a mean performance of 0.84 ± 0.13 (across six fault types). However, for the fault type ROFS, the multirobot system suffered a poor fault-detection performance, detecting the abnormally behaving robot only a proportion of 0.01 ± 0.02 of the time.

For the fault type ROFS, the low proportion of time that the faulty robot is detected is consequent to Unused-Device. The range-and-bearing sensors are not being used by the foraging robots during their search for resource sites, and in their return to the nest (*explore* and *return to nest* states in Fig 4a). Consequently, malfunctions in the range-and-bearing sensors do not affect the behavior of the faulty robot, and the faulty robot behavior is indistinguishable by the swarm, from explorer robots and robots returning to the nest.

### Robot swarm correctly tolerates normal swarm behaviors

**Fig 6:**
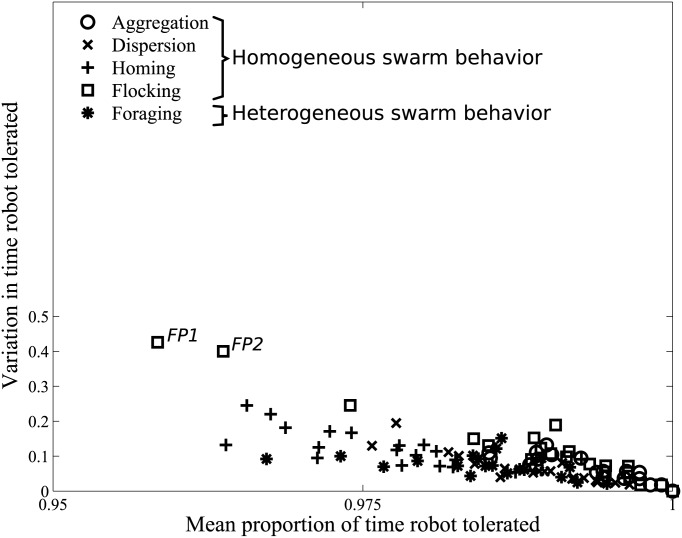
Tolerance to normal robot swarm behavior. Mean and variation in proportion of time robots tolerated, across the 20 robots of the multirobot system, in each of 20 replicates, and five normal behaviors: (A) aggregation (circles), (B) dispersion (crosses), (C) homing towards stationary landmark (pluses), (D) flocking (squares), and (E) cooperative foraging (asterisks).

The success of a fault detection system depends as much on its capacity to avoid false positives and correctly classify normal swarm behaviors, as its capacity to detect the faulty robots. Consequently, we evaluated our fault detection algorithm in a series of experiments in which all 20 robots behaved normally, performing homogeneous and heterogeneous swarm behaviors. We measured the proportion of time the robots were correctly classified by the multirobot system as behaving normally (tolerated). In Fig 6, we have plotted the mean proportion of time that robots are tolerated in each of 20 replicates of the five normal swarm behaviors (horizontal axis), and the variation between the 20 robots observed in each replicate calculated as the difference between the maximum and minimum time tolerated (vertical axis).

The mean proportion of time that normally behaving robots were correctly classified was high across all experiments, at 0.99 ± 0.01 for aggregation, 0.98 ± 0.01 for dispersion, 0.99 ± 0.01 for flocking, 0.97 ± 0.01 for homing, and 0.98 ± 0.01 for cooperative foraging (Mean±SD). The variation in time tolerated between the robots of the swarm, in individual replicates, is low (less than 0.25), in all but two replicates of the robots performing the flocking behavior (see false positive *FP1* and *FP2* in Fig 6). In these two replications, the false positives were consequent to one to five robots taking longer to join the flock (the particular robots random walked between 40 s and 120 s before they encountered the flock; all the other robots had initiated flocking by 30 s into the experiment). The detected robots thus were, in fact, behaving abnormally, consequent to their initial positions in the swarm, and not because of any faults on the robots.

### Fault detection unaffected by dynamic transitions in robot swarm behaviors

A principal advantage of our fault-detection algorithm is that the classification of normal/abnormal behaviors, for the detection of the faulty robots, is continually learned online while the robots operate in the task environment. Therefore, our fault-detection system has the capacity to adapt to changes in the normal robot swarm behaviors, while avoiding the misclassification of normal behaviors as faulty. The minimization of false positive incidents is essential, as the subsequent fault diagnosis and fault accommodation procedures may not only be costly, but could lead to the exclusion of capable robots from the multirobot system.

**Fig 7:**
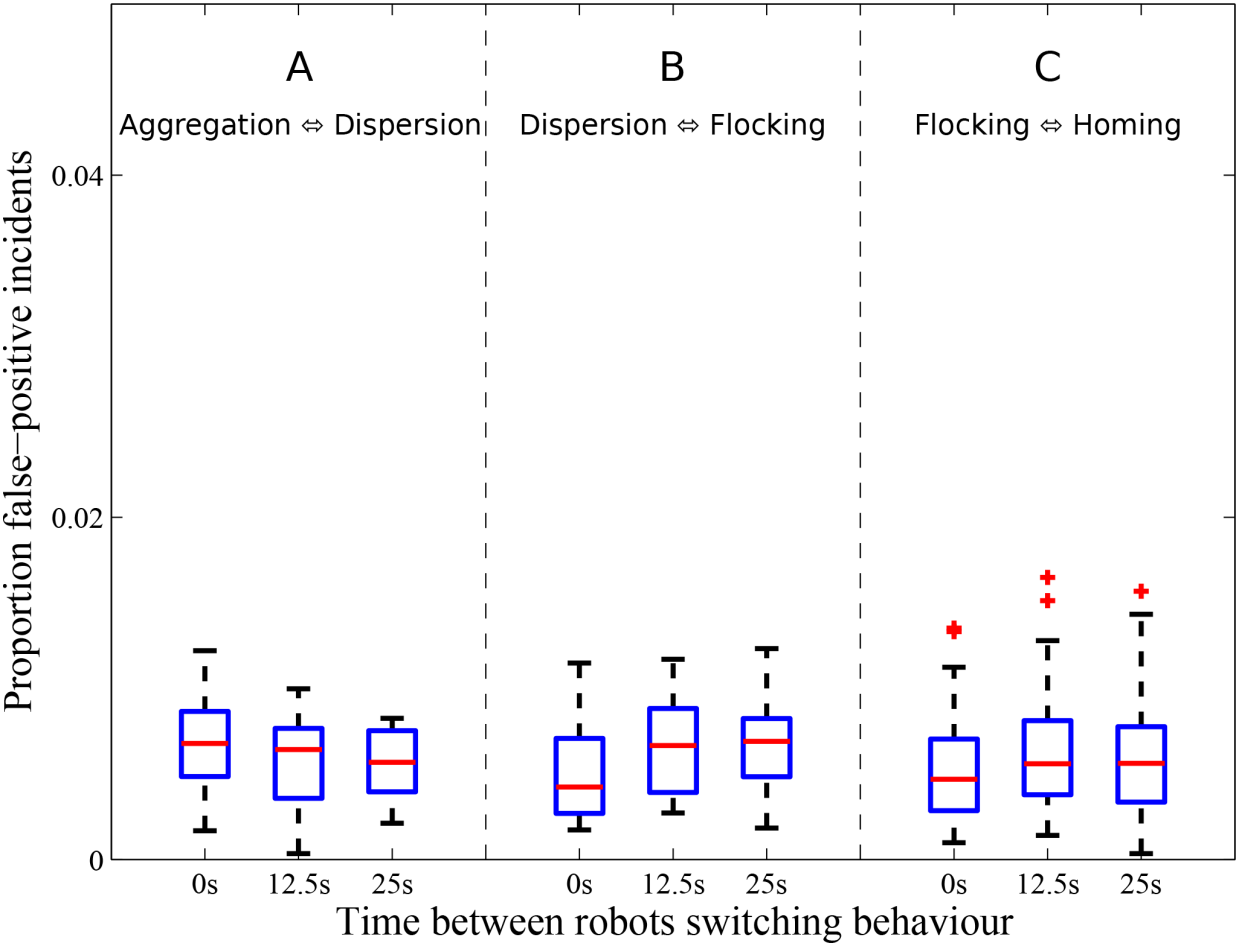
Tolerance to changes in normal robot swarm behavior. Proportion of false-positive incidents across the 20 robots of the multirobot system, in each of 20 replicates, and two transitions in normal behavior: (A) aggregation to dispersion to aggregation, (B) dispersion to flocking to dispersion, and (C) flocking to homing to flocking. Robots transitioned between the swarm behaviors in intervals of 0 s (instantaneously across the swarm), 12.5 s, and 25 s, in three separate and independent experimental setups.

We setup a series of experiments to assess our fault-detection system’s capability to avoid false positives when transitions in normal behavior occurred over time. We conducted 20 replicates in which all robots switched behavior. Each experimental replicate had a duration of 1,500 s. In each replicate, the robots started out by performing a particular normal behavior for the first 500 s, then gradually switched to a second behavior within the next 500 s, and finally reverted back to their original behavior within the remaining 500 s of the experiment. The fault-detection system was evaluated with three different combinations of behaviors: (i) aggregation to dispersion to aggregation, (ii) dispersion to flocking to dispersion, and (iii) flocking to homing to flocking. Robots were selected at random (following a uniform distribution) to switch their behavior, and the time between robots switching behavior was 0 s (behavior transitioned instantaneously across swarm), 12.5 s, and 25.0 s (in three separate and independent experimental setups, for each of the behavior combinations). The results, in terms of the proportion false-positive incidents, for the different behavior combinations and transition periods are shown in Fig 7.

Our fault-detection system achieved a low number of false-positive incidents for all three behavior transition combinations evaluated. The mean proportion of reported false-positive incidents sustained by the multirobot was no more than 0.01 for the aggregation–dispersion, dispersion–flocking, and flocking–homing behavior transition combinations. Additionally, the proportion of false-positive incidents incurred by the multirobot was not affected by the robot swarm behavior switching times (Kruskal-Wallis test: number of false-positive incidents not significantly different from each other, all *p* > 0.1).

### Fault detection resilient to perturbations in the robot swarm environment

**Fig 8:**
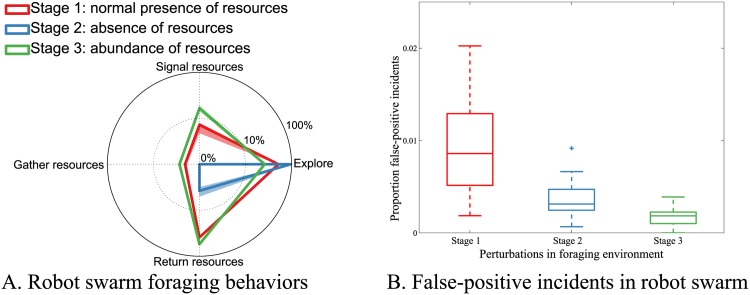
Tolerance to perturbations in the foraging environment. The robots were first perturbed to a foraging environment comprising an absence of resources (Stage 1 to Stage 2), and subsequently to an environment with an abundance of resources (Stage 2 to Stage 3): (A) Median ± IQR of robots exhibiting distinct behaviors of exploration, signaling, retrieval of resources at foraging site, and returning foraged resource to the nest, all during cooperative foraging, and (B) the proportion of false-positive incidents across the 20 robots of the swarm, in each of 20 replicates.

Our fault-detection system’s capability to avoid false positives was also assessed when normal behavior transitions were consequent to perturbations of the swarm’s task environment. The foraging case study (Fig 3) was selected for these experiments, as the robots of the swarm are required to adapt to changes in their environment. We conducted 20 replicates in which the foraging environment was perturbed. Each experimental replicate had a duration of 3,000 s, and comprised of three stages. In Stage 1, the foraging resource site was randomly located in the 3 × 3 m^2^ arena (Fig 3a), at least 2 m from the nest site. Subsequently, in Stage 2, the resource site was removed from the environment, to represent a scarcity of resources. In Stage 3 of the experiment, the resource site was quadrupled in size, and placed no more than 10 cm from the nest site, to represent an abundance of resources. Each of the three stages lasted for 1000 s, to make sure all the robots of the swarm had adapted their behavior to the perturbed foraging environment. The number of robots allocated to different foraging behaviors in each of the three foraging environments, and the proportion false-positive incidents, are shown in Fig 8.

In our experiments, perturbations to the foraging environment significantly affected the number of robots allocated to exploration, signaling, resource retrieval and transportation to the nest (Kruskal-Wallis test, *p* < 0.001 for all four behaviors; Fig 8a). The transition to an environment with an absence of resources (Stage 1 to Stage 2) resulted on average in a 81.5% increase in the number of explorer robots (searching for resources), a 89.4% decrease in the number of robots returning foraged resources to the nest, and was accompanied by a complete absence of robots signaling new resource locations, and retrieving further resources at foraging sites. The subsequent perturbation to an environment with an abundance of resources (Stage 2 to Stage 3) translated into a 73.2% decrease in explorer robots, and a 12.4% increase in robots returning foraged resources to the nest. Furthermore, in this environment, 16.7% and 2.8% of the robots in the swarm exhibited signaling and resource retrieval behaviors, respectively. In summary, the robots of the swarm allocated themselves to different behavior roles in consequence to the nature of the perturbation in their environment.

Our fault-detection algorithm achieved a low number of false-positive incidents in all three sequentially evaluated foraging environments (in Stage 1, Stage 2, and Stage 3, mean proportion of false positives no more than 0.01, see Fig 8b). In perturbing the foraging environment by removing resources (Stage 2), the robots accumulated a slightly higher proportion of false-positive incidents at 0.03 ± 0.02 (Mean±SD) during the behavior transition time-window immediately following the perturbation (Kruskal-Wallis test while correcting for size of transition time-window of 140 s for robots to transport already foraged resources to the nest, *p* < 0.001). However, environment perturbations did not always translate to an increase in false-positive incidents. The subsequent perturbation to an abundant resource environment (Stage 3) registered a negligible proportion of false-positive incidents at 0.001 ± 0.003 in the behavior transition time-window (required to locate the newly introduced abundant resource site, estimated empirically at 50 s) following perturbation. In summary, our fault-detection system is largely resilient to environment changes. While some environment perturbations translate to an increase in false-positives, the number of such incidents remains minuscule—even during such perturbations.

### Importance of different algorithmic components on robot swarm performance in fault detection

The robot swarm in our experiment relies on the following three distinct algorithmic components to perform fault detection: (i) Phase A—robots of the swarm estimate the behavioral features of their neighbors, and subsequently employ an inter-robot voting scheme to select the most popular feature values for each observed robot; (ii) Phase B—every robot detects abnormally behaving neighbors, based on a simple majority on normal/abnormal behavior classifications of its CRM accumulated over a series of consecutive control cycles; and (iii) Phase C—the robots form voting coalitions to consolidate their individual-level decisions on the detected behavioral abnormalities. We assessed the importance of each of these three phase on the fault-detection performance. Our results, detailed in Section B in S1 File, reveal that all three phases of our implemented fault-detection system are necessary for the swarm to achieve good performance. By employing our system, the robot swarm is not only capable of accurately detecting faulty robots in the swarm, but is also able to avoid false positives in classifying normal/faulty behaviors, despite changes in normal behavior, and perturbations in the swarm’s task environment.

## Discussion

In this study, we presented a decentralized exogenous fault-detection system for robot swarms. The process of fault detection follows three phases: (i) behavior observation, wherein robots of the swarm observe the behaviors of their neighbors over a period of time, subsequently encoding their observations as binary feature vectors; (ii) behavior classification, wherein an instance of the CRM [19] is run by every robot of the swarm to classify observed behaviors as normal/abnormal. Abnormalities are a symptom of faults in the observed robot; and (iii) coalition formation, robot consolidate their individual decisions on the classified abnormal behaviors into a swarm-level decision on the identity of the faulty robots.

Obtaining reliable inter-robot behavior observations in a distributed manner, the first phase of our fault-detection process, is challenging in large-scale swarms, but crucial for accurate fault detection. Robots observe the behaviors of their neighbors, using noisy sensors with limited range, essentially constraining the observations to be brief and sporadic. In taking advantage of the large number of robots in a swarm, multiple independent observation sources of the robot’s behavior improves the robustness in the estimated behavioral features. In our experiments, the range-and-bearing sensors onboard the e-puck robot are employed for behavior observation. The range-and-bearing sensors are readily available on many swarm robot platforms (e.g., see extension modules for Khepera III [36], eye-bot [37], foot-bot [38] and e-puck robots [39]). In the absence of a dedicated range-and-bearing sensor, time-division-multiplexed IR proximity sensors may provide the robots with equivalent sensor readings [40]. Alternate observation sensors such as popular onboard camera sensors may also be used for behavior observations, assisted by fiducial markers [41, 42] tagged on the robots of the swarm [43].

In the second phase of our fault-detection process, the classification output of the CRM abnormality detector is accumulated over a series of consecutive control cycles, prior to consensus formation on the detection of the faulty robot. Our results revealed that an increase in the length of the accumulation time window always translated into a lower number of false-positive incidents, accompanied however by a higher latency in the detection of faulty robots. The cost of accommodating a faulty robot is an important consideration in deciding the length of this accumulation time window. Scenarios wherein fault accommodation is expensive, and the immediate detection of faulty robots is not crucial to success of the swarm’s mission, may benefit from a high time window. By contrast, in critical tasks where faulty robot behavior may be catastrophic, the length of the accumulation time-window may be set to a relatively low value. While the fault accommodation cost may be valued based on prior knowledge of the swarm’s environment [44, 45], the possibility of dynamically costing fault accommodation remains an open question.

In our simulations, every robot of the swarm is synchronized in their fault-detection cycles. However, as local synchronization between robots in close proximity is sufficient for our distributed approach to fault detection, synchronization may easily be enforced in experiments with real robots (e.g., see synchronous flashing of fireflies [46], and bacterial quorum-sensing [47] applied to distributedly synchronize robot swarms [13, 48–50]). Furthermore, in our study, a relatively low degree of desynchronization of the robots internal clock did not severely impact the swarm’s performance in fault detection. In preliminary experiments (detailed in Table A in S1 File), a random perturbation of every robots internal clock following N(5 *s*, 2.5 *s*) resulted in the faulty robot continuing to be detected in most experiment replicates (Mean ± SD proportion of replicates, Sync.: 0.9 ± 0.2, De-sync.: 0.8 ± 0.2, Kruskal-Wallis test, *p* < 0.001), requiring a relatively higher time latency for fault detection (Sync.: 51.4 ± 18 s, De-sync.: 126.8 ± 35 s, *p* < 0.001). The impact of a more severe desynchronization of the robots on the performance of the swarm in fault detection is to be studied further.

A multi-layered approach to fault detection [7] integrates both endogenous [3–6] and exogenous fault detection [8–10, 12–14, 18, 51] in their design, consequently benefiting from both approaches. Robots employing a multi-layered approach can utilize the multitude of robots in large-scale swarms for exogenous fault detection, while simultaneously relying on endogenous fault detection when isolated, or in scenarios where a faulty robot’s neighbors may compensate for its abnormal behavior (see Table 4, SWARM-COMPENSATE fault-negative incidents). With a robot behavior observation model already in place, our exogenous fault-detection system may be easily extended with the inclusion of an endogenous fault-detection component. Such a component would allow robots to detect faults endogenously by comparing proprioceptively computed behavioral feature vectors, with behavioral feature vectors communicated by observing neighbors. In utilizing such an approach, false-negatives incidents due to the robot swarm compensating for abnormally behaving robots, would be avoided.

## Conclusions

Robot fault detection and fault tolerance represent two of the most important problems in the field of multirobot systems. Robot swarms in many real world scenarios, operating in unstructured environments for instance, require a fault-detection system that can adapt to temporal variations in the robots behavior and perturbations to the environment [52, 53]. The fault-detection system presented in this study demonstrates these capabilities. Namely, our fault detection system provides the following significant contributions for robot swarms:

- Distributed behavior observation model, which is resilient to sensory noise and intermittent and sporadic robot observations. Unlike previous work (e.g., [7]), our robots do not have to interrupt their task to observe each other for fault detection.
- Normal/faulty behavior-classification system [19] which can be tuned to balance the trade-off between latency in faulty detection and the number of false-positive incidents.
- A consensus algorithm to transcend normal/faulty decisions made by individual robots to a robust swarm-level decision on the status of the robot. Such a robust collective decision is critical to extend our algorithms to provide fault-recovery strategies for a robot swarm.
- Our resulting behavior-driven fault detection system operates independently of the controller architecture employed to execute the swarm behavior.

While our experiments have been performed in simulation, largely due to the high cost of individual e-puck robots, noise has been added to simulate the degree of stochasticity inherently associated with real robots. In ongoing work, we are investigating the deployment of our fault-detection system on a relatively small swarm of real mobile robots, and the potential for using our fault-detection system in more challenging outdoor scenarios.

## Supporting information

S1 File Electronic supplements of robot swarm simulation souce code and the ablation analysis of the fault-detection system. (PDF)
