# Supplementary material for: Generic, scalable and decentralized fault detection for robot swarms
Source: PLoS One. 2017 Aug 14;12(8):e0182058. doi: 10.1371/journal.pone.0182058 (PMC5555700; doi:10.1371/journal.pone.0182058)
Supplement: S1 File — (PDF) [file pone.0182058.s001.pdf]

Electronic supplement
Generic, Scalable and Decentralized Fault Detection for Robot Swarms

Danesh Tarapore[1,2], Anders Lyhne Christensen[3,4,5], Jonathan Timmis[2]

[1] School of Electronics and Computer Science, University of Southampton, Southampton SO17 1BJ, U.K.
[2] York Robotics Laboratory and the Department of Electronics, University of York, Heslington, York YO10 5DD, U.K.
[3] Bio-inspired Computation and Intelligent Machines Lab, 1649-026 Lisbon, Portugal
[4] Instituto Universitário de Lisboa (ISCTE-IUL), 1649-026 Lisbon, Portugal
[5] Instituto de Telecomunicações, 1049-001 Lisbon, Portugal

A Robot swarm simulation

The robot swarm simulation source code, including the implemented homogeneous (aggregation, dispersion, flocking and homing) and heterogeneous (cooperative foraging) normal swarm behaviors, the robot fault-injection system, and the algorithms for fault detection, are all available for download at `https://github.com/daneshtarapore/tarapore_2017_plosone`.

B Importance of different algorithmic components on robot swarm performance in fault detection

The robot swarm in our experiment relies on the following three distinct algorithmic components to perform fault detection: (i) Phase A – robots of the swarm estimate the behavioral features of their neighbors, and subsequently employ an inter-robot voting scheme to select the most popular feature values for each observed robot; (ii) Phase B – every robot detects abnormally behaving neighbors, based on a simple majority on normal/abnormal behavior classifications of its CRM accumulated over a series of consecutive control cycles; and (iii) Phase C – the robots form voting coalitions to consolidate their individual-level decisions on the detected behavioral abnormalities. Below, we assess the importance of each of these three phase on the fault-detection performance. The Table A lists the details on the experimental setups used.

Phase A: Voting on observed feature vectors

In the assessment of Phase A of our fault-detection algorithm, we analyzed the importance of the inter-robot voting scheme employed by individual robots for the robust estimation of observed

Table A: **Experimental setups to assess the importance of the different phases of fault detection**

Setup	Description
(I)	**Detection of the faulty robot:** A single robot in a swarm of 20 robots exhibits a fault. The fault-simulating behaviors considered are PMIN, PMAX, PRND, ROFS, LACT, RACT, and BACT. The normal behaviors for the remaining 19 robots of the swarm are, aggregation, dispersion, flocking, homing, and cooperative foraging, resulting in a total of 35 combinations, each replicated 20 times.
(II)	**Tolerance (minimization of false-positive incidents) to normal robot behaviors:** All 20 robots of the swarm behave normally. The normal behaviors considered are, aggregation, dispersion, flocking, homing, and cooperative foraging, in five separate and independent experiments, each replicated 20 times.

behavioral features. A modified fault-detection algorithm having no voting scheme on behavioral feature values was evaluated for its capability in detecting the faulty robots of the multirobot system (Setup (I) in Table A). In the absence of inter-robot voting on observed features, the robots of the multirobot system detect the faulty robots based solely on their own individual behavioral observations.

Experimental results support the importance of sharing observed behavioral information between robots of the multirobot system to robustly estimate behavioral feature values: in the absence of voting on observed behavioral information, the multirobot system suffered a significant decrease in the proportion of time the faulty robot was detected (Mann-Whitney test: $p < 0.001$, sample size of 35 normal/faulty behavior combinations $\times$ 20 replicates, $df = 1398$). The median proportion of time the faulty robot was detected was 0.82 and 0.67, with and without voting on observed feature vectors, respectively. Additionally, the latency in the detection of the faulty robot from the time the fault occurred, also differed (Mann-Whitney test: $p < 0.001$, $df = 1398$), at 34.1 s and 84.2 s, with and without voting on observed feature vectors, respectively.

Phase B: Integrating CRM output over consecutive control cycles

In Phase B of our fault-detection algorithm, each robot employs the CRM-based normal/abnormal behavior classifier on the observed behavioral feature vectors of its neighbors. For a reliable detection of the abnormally behaving robots, in our experiments, the CRM output is accumulated over 90 consecutive control cycles, and subsequently employed as a simple majority by each robot to classify the behavior of its neighbors. To assess the importance of this accumulation of behavior classifier output, we performed the experiments (I) and (II) (see Table A), in each of which the CRM output was accumulated over a series of 1, 40, 90, 140, and 190 consecutive control cycles, in separate and independent experiments. The results, in terms of the number of false-positive incidents, and the latency in the detection of faulty robot behavior, for the different accumulation periods are shown in Fig. A.

Whatever the behaviors exhibited by the multirobot system, longer accumulation time windows always translated into decreased number of false-positive incidents, and simultaneously an increased latency in fault detection (Kruskal-Wallis test, both $p < 0.001$; Fig. A). When the fault-detection algorithm employed an accumulation time window of a single control cycle, the multirobot system was inflicted with 153.4 ± 234.7 (Mean $\pm$ SD) false-positive incidents and required a latency of $29.1 \pm$

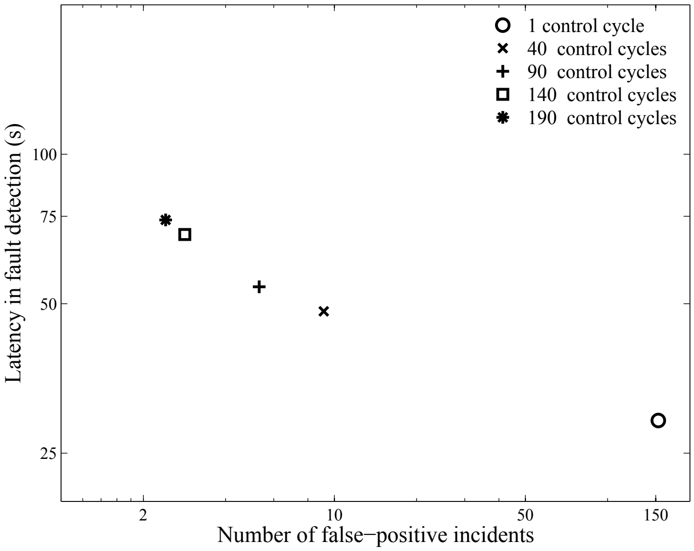

Fig A: **Trade-off between tolerance to normal robot swarm behavior, and the latency in the detection of faulty robot behavior.** Performance in fault detection for differences in the implementation of Phase B of the fault-detection algorithm; every robot detects its abnormally behaving neighbors, based on a simple majority on normal/abnormal behavior classifications of its CRM accumulated over a series of 1, 40, 90, 140, and 190 consecutive control cycles, in separate and independent experiments. Each point indicates for a given accumulation time window, the mean number of false-positive incidents (horizontal axis), across the 20 robots of the multirobot system, in each of 20 replicates, and five normal behaviors, and the mean latency (vertical axis) in the detection of faulty robot behavior across the 35 normal/faulty behavior combinations, for each of 20 replicates.

16.2 s to detect faulty robot behavior. By contrast, an accumulation time window of 190 control cycles translated into a considerable decrease in false-positive incidents at 2.4 ± 6.4, accompanied by more than twice increase in the latency for fault detection, at 73.7 ± 27.3 s (Mann-Whitney test, $df = 38$, both $p < 0.001$).

Phase C: Swarm coalition formation on detected abnormal behavior

In the final phase of our fault-detection algorithm, the robots consolidate their individual-level decisions on the detected behavioral abnormalities in their neighboring robots into a swarm-level decision on the normal/abnormal state of the robots. Such an inter-robot coalition forming process prevents false positive incidents consequent to misclassification by a single or few robots of the swarm. To assess the importance of swarm coalition formation, we eliminated Phase C from our fault-detection algorithm. Consequently, in our modified fault-detection algorithm, robots of the swarm determined, independently and separately, the normal/abnormal state of their neighbors. The capability of the modified fault-detection algorithm to minimize false-positive incidents was evaluated in Setup (II) (Table A).

Our fault detection results suggest the significance of consolidating individual-level decisions on

the detected behavioral abnormalities. In the absence of the swarm coalition formation process, the multirobot system experienced a difference in the number of false-positive incidents (Mann-Whitney test: $p < 0.001$, sample size of 5 normal behaviors $\times$ 20 robots $\times$ 20 replicates, $df = 3998$). In the presence of the swarm coalition formation process, the number of false-positive incidents incurred by the multirobot system was 5.3 ± 12.2 (Mean $\pm$ SD across the 20 robots of the multirobot system, in each of 20 replicates, and five normal behaviors). By contrast, without coalition formation, the number of false-positive incidents incurred by the multirobot system was considerably higher at 252.6 ± 371.6.

In summary, all three phases of our implemented fault-detection system are necessary for the swarm to achieve good performance. By employing our system, the robot swarm is not only capable of accurately detecting faulty robots in the swarm, but is also able to avoid false positives in classifying normal/faulty behaviors, despite changes in normal behavior, and perturbations in the swarm's task environment.
